# How Does the Horizontal Position of Pictures and Text Affect Product Evaluation? Based on Left and Right Position Effect

**DOI:** 10.3389/fpsyg.2022.841480

**Published:** 2022-07-11

**Authors:** Zan Huang, Yingjue Du, Feifei Xu, Chuming Hu

**Affiliations:** ^1^School of Management, Jinan University, Guangzhou, China; ^2^Research Institute on Brand Innovation and Development of Guangzhou, Guangzhou, China

**Keywords:** image display, text declaration, left-right position, psychological closure, product attributes, product evaluation

## Abstract

Due to the untouchability of online shopping environment, image and text description, as two main ways of product information display, are important indicators for consumers to evaluate products. However, few studies have discussed the synergistic effects of image and text information on consumers. In the present study, in conjunction with the left-right position effect, we examine the expectation that horizontal placement of visual stimuli in different directions has a strong influence on consumers’ product evaluation preferences. This implicit assumption is based on consumers’ unconscious psychological need for closure when processing information. The authors conducted three studies to investigate the relative effects of image information and text statements at different locations in online shopping pages on consumer product evaluations. The results show that: (1) when the evaluation object is a search product, compared with the display mode of left text-right image, the display mode of left image-right text plays a more significant role in consumer product evaluation. The results of experiential products were just the opposite. The way of presenting the text declaration on the left and image on the right has a stronger impact on consumers’ evaluation preference for experiential products (Study 1 and Study 3). (2) The difference in consumers’ evaluation mode of different presentation sequences based on product attributes is driven by their visual information processing fluency (Study 2). These preferences are robust, and it is worth noting that only the order of graphic presentation has no significant influence on consumer product evaluation preference.

## Introduction

Along with the popularity of the Internet, more and more consumers are preferring to make purchases in e-marketplaces. E-mall mainly uses pictures and text information to show consumers the characteristics of goods. However, compared with traditional offline shopping malls, e-malls are virtual and cannot provide consumers with a realistic experience of goods. Undoubtedly, this will impede consumers’ assessment and judgment of commodity attributes and reduce the likelihood of actual purchase behavior occurring (Xia et al., 2020). In particular, for experience attributes, it is difficult to achieve the ideal display effect with the display of goods across the screen. In order to compensate for the lack of physical sensory experience, online platform merchants need to provide rich and accurate visual and linguistic descriptions of product information. Online visual information is presented in a variety of ways. Previous studies have explored the influence of visual cues based on images and texts on consumers’ cognition, emotion and purchase intention of product evaluation in online environment (Pieters and Wedel, 2004; Orús et al., 2017), but little attention has been paid to the effect of left and right position information of images and texts.

In addition, product type is an important dimension to study consumer behavior and marketing stimulus. Regardless of product type, purchase decisions involve trade-offs related to product attributes (Kim, 2017). Marketers can manipulate text descriptions to reflect search attributes and experience attributes separately, even for the same product. For example, when buying a laptop, the search tagline is described as “… Notebook new, 14 inch gold size screen, 8 G memory, 4 G independent graphic card, net weight 2 Kg, the king of high-end machine cost performance….” The experiential advertising slogan is described as “… Game online no lag, light and portable, anytime, anywhere to enjoy the ultimate audio-visual game experience….” The former uses objective and specific language or specifications to describe product quality information, so consumers can accurately evaluate the attributes of products even if they do not buy or try them. The latter mainly relies on consumers’ previous experience or association with relevant attributes to evaluate products (Ford et al., 1990; Blankson and Kalafatis, 1999; Srinivasan and Till, 2002). In the context of online purchase, consumers have different logics and processes for information processing of search items and experiential items (Nelson, 1970; Jain and Posavac, 2001). Our study attempted to investigate the following two key questions to fill in the past research gaps:

1. Will the different horizontal presentation positions of images and texts affect consumers’ product evaluations?
2. Which kind of graphic information presentation is more suitable for search items and experiences (left image-right text vs. left text-right image)?

To answer these research questions, we conduct an empirical study in the context of China’s e-commerce market. Based on the theory of psychological closure, this paper explains the interaction mechanism of different product attributes and graphic position presentation (left image-right text vs. left text-right image) on product evaluation.

## Literature Review and Research Hypotheses

### Presentation of Left-Right Position of Products and Consumers’ Reaction

Images and texts are the basic elements in consumer information environment. As two carriers to present product information to people, pictures and texts have different characteristics. The former can be used as a simulation of real objects, showing the details of actual products. While the latter is composed of many words. Every word represents a kind of thing, which has a wider scope. Words can grasp the essential characteristics of things and omit trivial details. Previous studies have reported the important role of product information in consumers’ purchasing decisions (Mitchell and Boustani, 1994; Kim and Lennon, 2008), and focuses on the influence of graphic visual information on consumers’ attention (Pieters and Wedel, 2004; Pieters et al., 2007; Peschel and Orquin, 2013), memory (Shepard, 1967; Houston et al., 1987; Noseworthy et al., 2011), attitudes and decisions (Hirschman, 1986; Hoegg et al., 2010).

There are different opinions on the functions of picture and text information. For example, compared with the text information, the picture information in the advertisement can stimulate the positive emotion of the subjects, and then affect their product evaluation and purchase intention (Mitchell and Olson, 1981). However, contrary research results show that both images and text information in advertisements will affect people’s emotions and cognition, but only text information will significantly affect consumers’ purchase intention (Kim and Lennon, 2008). Consumers rely more on words to make decisions, and picture information generally exists only as evidence of choice. In addition to the comparison between picture information and text information, some scholars also pay attention to whether the interaction between the two can produce better consumer response. For example, the presentation of different types of images and text information can affect consumers’ interaction with brands and their relationship with brands to varying degrees. When the display of image information and text information is consistent, consumers’ brand participation behavior can be more stimulated (Liu et al., 2018). Wyer and Adaval (2009) found that when consumers evaluate information containing both images and text, advertisers vacillate between image-centric and text-centric advertising strategies (Wyer and Adaval, 2009).

Most of the previous studies only examined the single attribute of the product, which may produce different results in different decision-making environments, resulting in different effects of image and text information in marketing communication. Moreover, there is no unified explanation of the impact of the two on product evaluation. Different from previous studies that emphasized object processing, this study focuses on the impact of left-right horizontal display order on product evaluation. Three-dimensional space theory shows that front and back, left and right, up and down together constitute three-dimensional space dimension. In the real online shopping situation, product graphic information will also be staggered in horizontal and vertical order. This study mainly focuses on the influence of horizontal left and right position order.

People have lateral bias from left to right when processing visual information (Christman and Pinger, 1997). On the one hand, left-to-right orientation is determined by the neural coding system. According to hemispheric processing theory, people tend to represent events and process visual information from left to right because the hemispheres guide spatial attention from left to right as the brain processes information, and this left-to-right orientation still exists during information encoding (Chatterjee et al., 1999; Chatterjee, 2001). On the other hand, people’s cultural learning connections between time and space determine the existence of directionality from left to right.

In many cases, the processing of visual information invokes the use of unconscious metaphors by consumers to relate the target domain (e.g., left and right location) to another source domain (e.g., time) (Forceville, 2002). For example, driven by daily practice or reading habits, individuals in left-leaning reading cultures will move their attention from left to right when they recognize objects such as words, symbols or charts, and their eyes will recognize objects faster from left to right (Spalek and Hammad, 2005). This visual cognition leads people to believe that events start from the left side of the page and end from the right side (Santiago et al., 2007). Abundant stimuli shift visual attention and increase the attentional representation of objects that start from the left and advance to the right.

Other scholars have reported similar views and extended the concept, showing that left and right positions are conceptually related to beginning and end or past and future time (Ouellet et al., 2010). Chae and Hoegg (2013) reported the left and right in the past and future time attribute is applied to the product advertising, when consumers see the product position and time metaphors consistent advertising, their products have a better attitude, in particular, modern lamp placed in the picture on the right, antique lamps placed in the picture on the left, the consumer response is better (Chae and Hoegg, 2013). These studies suggest that left and right positions inspire temporal associations. We believe that this association comes from the visual cognition of people, that is, the left side of position is associated with the past/beginning/early concept of time, while the right side is associated with the future/end/late concept of time, which reflects a visual cognitive representation (Vallesi et al., 2008). It is worth noting that this study focuses on left-leaning reading habits, while special right-leaning groups, such as Arabs, whose reading and writing habits shift from right to left, are not covered by our discussion.

### Interactive Influence of Product Attributes and Left-Right Graphic Position on Consumer Product Evaluation

Cognitive psychologist believes that psychological closure is the basic cognitive needs of human beings. It’s a state that people want to achieve, where they feel the completion of an experience and give a sense of the “past” when recalling life events. People desire to complete events (Kivetz et al., 2006; Nunes and Dreze, 2006; Sevilla and Kahn, 2014), and achieve this goal through other means. For example, the physical act of shutting down (putting a painful narrative of an emotional relationship in an envelope) helps people achieve psychological closure over emotional experiences (Li et al., 2010). The unfinished event will make people feel uneasy and uncertain psychologically (Beike and Wirth-Beaumont, 2005; Beike et al., 2007), resulting in negative emotions. In order to avoid negative emotions, people will have different psychological closure needs in different situations. For example, when people want to quickly understand and use information, they tend to have a higher need for closure (Dechesne and Kruglanski, 2004). We propose that there is an interactive effect of the position of image and text information and the type of product attributes on the psychological closure of information processing, which in turn affects the consumer’s evaluation of the product. This is because the information positions (left vs. right) will lead to different levels of psychological closure needs; while, the form of information (picture or text) and the type of product attributes interactive influence the degree of divergence of people’s minds.

Firstly, the information positions (left vs. right) will lead to different levels of psychological closure needs. In the stage of consumer information processing, the left side means the beginning of information processing. By contrast, the right side means the end of information processing (Finke, 1995). We infer that, it is the left side shows the abstract content to help priming consumers’ divergent thinking and cognitive status is non-closed. However, the concrete content presented on the right side can answer consumers’ cognition of information and meet their psychological needs for closure.

Secondly, the form of information (picture or text) and the type of product attributes interactive influence the degree of divergence of people’s minds. Product attributes type have different effects on consumer psychology, emotion, information processing mode and behavior, which are central to product evaluation and preference formation (Lancaster, 1971). Generally, product attributes can be classified into two categories: search attributes and experience attributes (Huang et al., 2009). Search attributes refer to the information that can be obtained without purchasing or using the product, such as the color of the chair or the number of calories, ingredients and price, etc. These attributes reflect the objective quality of the product. Experience attributes are generated from subjective experience and can only be verified by the use of a product, such as the “user-friendliness” of a computer or the sharpness of a photo taken by a mobile phone (Blankson and Kalafatis, 1999; Srinivasan and Till, 2002).

Compared to images, text descriptions of experience attributes are considered a higher level, more abstract visual effect, and the text of experience attributes on the left can improve the divergence of consumers’ thinking. While the picture on the right shows why the effect of text description on the left can be achieved, which helps to converge divergent thinking and ends consumers’ cognition of information (Huang et al., 2017). Under the condition of left image-right text, the product information displayed in the left picture is more abstract, while the text reflecting search attributes on the right helps consumers obtain more useful information about quality (Wright and Lynch, 1995), which ends consumers’ cognition of information and meets their psychological closure needs. Therefore, in the left image-right text presentation, consumers will be more favorable to the evaluation of products with search attributes. When the text reflects the attributes of experience, it reflects the subjective experience and personal feelings of consumers. Since consumers cannot directly contact commodities in the Context of the Internet, consumers’ imagination of relevant experience will be initiated. Such imagination ability is believed to benefit from abstract thinking (Ward, 1995), and leads to the divergence of people’s thinking. Therefore, the presentation mode of the picture on the left and right is more conducive to improving consumers’ evaluation of the product of experience products. Based on this, we propose the following hypothesis:

H1: When the product text is described as a search attribute, the presentation of the left image-right text leads to higher product evaluation than the presentation of left text-right image;

H2: When the product text is described as an experience attribute, the presentation of left text-right image leads to higher product evaluations than the presentation of left image-right text.

### Mediating Role of Information Processing Fluency

People evaluate objects based on subjective feelings of ease or difficulty (Schwarz, 2004). When people are faced with products that conform to their behavior habits or value orientation, they are more likely to stimulate their representation of goals or habits, and then carry out smooth processing of products. Lee and Labroo (2004) pointed out that when the information framework is consistent with the individual’s natural way of thinking, the information may be easier to process, and the subjective experience of fluent processing will in turn affect the subsequent evaluation, improve the persuasiveness of information, and thus generate a better attitude (Lee and Labroo, 2004).

There are two main reasons why the fluency of information processing affects consumers’ product evaluation. On the one hand, according to the fluency-emotion connection model, high fluency in the process of processing information can lead to positive emotions. Although such emotions are transient and weak, they become important clues for subsequent cognitive evaluation and judgment. Thus, individuals are more positive in evaluating the processed objects (Winkielman and Cacioppo, 2001). On the other hand, when people process information smoothly, they feel that their actions are correct, and then the behavioral tendencies associated with them are enhanced (Avnet and Higgins, 2006).

Information processing fluency is the ease or difficulty with which information can be processed, or the ease or difficulty with which content can be brought to mind (Schwarz, 2004). When consumers’ mental representation of goals is matched with the goal presentation model, such matching improves the difficulty of information processing, thus producing more favorable goal evaluation (Chae and Hoegg, 2013). Previous studies have reported similar views. For example, when the left-right presentation of healthy (vs. unhealthy) food is consistent with the mental representation of consumers, it is beneficial for information processing, thus increasing consumers’ preference for choosing healthier food (Romero and Biswas, 2016). In addition, consistency of time-related product presentation with time mental representation would improve information processing fluency and thus improve advertising product attitudes (Chae and Hoegg, 2013).

Based on the analysis above, whether it is a search attribute or an experience attribute, marketers can use a combination of images and text to present to consumers. However, the left and right position of images and text will affect the persuasive effect of the advertisement. This is because there is a matching effect between the presentation form of images or text and the position of information presentation. That is, the search information presented in the form of left image-right text and the experience attributes presented in the form of left text-right image are matched with consumers’ mental representations, respectively. It’s easier to process when the mental representation is consistent with the presentation form. Based on above, we propose the following hypothesis:

H3: The interactive influence of product attributes and left/right position presentation on consumer product evaluation is mediated by information processing fluency.

**FIGURE 1:**
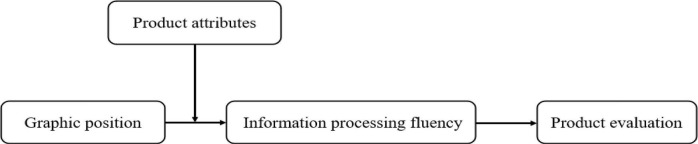
The research model.

The research model in this article is shown in Figure 1.

## Methods and Results

In the following three studies, we aim to reveal the interactive effects of different product attributes and left and right position presentation on product evaluation. The actual online purchasing behavior of consumers needs to go through several stages of product browsing, evaluation and purchase successively. Among them, product evaluation significantly affects the purchasing decision and behavior of products, but there are still other factors that affect consumers’ purchasing behavior. In order to reduce the interference of other influencing factors, we focused on consumer product evaluation and expressed product evaluation (interest degree, quality, trust, support, and usefulness) by 5 items based on previous research results (Peracchio and Tybout, 1996). All items were measured on a 7-point Likert scale ranging from “1 = strongly disagree” to “7 = strongly agree.” The selected products need to be constructed by consumers to contain different characteristics (Voss et al., 2003). In Study 1, we chose mobile phones as the target material, which is often considered to have both search attributes and experience attributes (Mokhlis and Yaakop, 2012), and investigated the influence of left-right position presentation of text and image on consumers’ reaction to products. In Study 2, we chose hoodies as experimental materials and observed the mediating mechanism of information processing fluency. In Study 3, we chose mountain bikes as the experimental product and tested the robustness of the findings.

### Study 1

#### Pre-test

In order to enable the experimental material cell phone can have both search and experience attributes, and to design the text stimulus material for the main study, the study used two cell (product attribute: search attribute vs. experience attribute) between-subjects design. The data were collected from 80 volunteers recruited on an online questionnaire site. Two of them did not report valid results and were excluded from the analysis, while the remaining 78 volunteers (33 men, *M*_*age*_ = 24.1, SD = 2.32) completed the study.

The introduction of the mobile phone includes not only features such as CPU, pixel, and appearance, but also experience features such as smooth use and clear photos. To measure participants’ perception of search attributes and experience attributes of the phone, participants were randomly assigned to one of two scenarios. Firstly, we explained the meaning of search attributes and experience attributes to the participants. Then, participants in the search attribute condition saw only the search attribute description, and participants in the experience attribute condition saw only the experience attribute description. They were then asked to report the product attribute description evaluations they saw using a single item (5-likert scale, 1 = search attribute, 5 = Experience attribute). In addition, in order to increase the robustness of the study, we chose the description of the appearance function and camera function of the mobile phone as experimental materials.

Independent-samples *t*-test showed that there was a significant difference between the search attribute and the experience attribute of the phone appearance perceived evaluation by participants (*M*_*search*_ = 1.85, SD = 1.29; *M*_*experience*_ = 3.51, SD = 1.47), *F*(1,76) = 4.01, *p* = 0.03, *Cohen’s d* = 1.2, there was also a significant difference in search attribute and experience attribute of camera function (*M*_*search*_ = 2.13, SD = 1.44; *M*_*experience*_ = 4.04, SD = 1.04), *F*(1,76) = 4.22, *p* = 0.03, *Cohen’s d* = 1.52. These findings indicate that our manipulation of experimental material search and experience attributes is successful.

Based on the pre-study results, mobile phones were selected as the stimulus material for the text statement of search attributes and experience attributes that met the research requirements. The text statement used in studies is shown in Table 1.

**TABLE 1:** Stimulus material in studies.

Study 1		
**Product Category**	**Attributes**	**Text statement**
Smart Phone	Search	6.53-inch HD Pearl screen, ultra-high screen ratio, blue and black curved glass body, 2244 × 1080 pixels, front 24 million pixels, rear 12 million + 16 million + 8 million pixels, AI handheld super night scene + AI smart anti-shaking, supporting 2.5 cm macro photography, Leica large wide Angle lens.
	Experience	Exquisite grip, gorgeous appearance, exquisite workmanship to create visual beauty, smooth arc, elegant and pure, love at first sight, multi-focus section switch, can be widely seen in the world, but also can see the micro, night shooting clearer, picture quality is more outstanding, AI photography master, let life more wonderful.
**Study 2**		
Sweatshirt	Search	The outer layer is 100% cotton, and the inner layer is made of warm fleece fabric, dyed with high-end gray dye and sewn with fine cotton thread, the outer layer is 100% cotton, and the inner layer is warm fleece fabric, printed with 0.1 mm precision, XS-XXL size is suitable for a wide range, mainly used for class parties.
	Experience	The cloth is soft and delicate, healthy and skin-friendly, bringing you baby-like softness, plush and thick, comfortable and warm, beautiful and practical, showing the quality of details, adopt classic color matching, fashion is versatile, simple and not lose the trend, “the more you grow, the more youthful” printing integration, highlight the youth color, let you enjoy a good time together.
**Study 3**		
Mountain Bike	Search	Rim size: 26 inches; product net weight: 15 kg; suitable height: 155–185 cm; the whole height of the car: 98 cm; seat height: 79–94 cm.
	Experience	Thickened anti-deformation frame, sturdy and durable; anti-slip and wear-resistant tires, facing various roads; thickened shock-absorbing front fork, comfortable and good riding; dual mechanical disc brakes front and rear, convenient and safe; positioning flywheel, accurate and easy.

#### Stimulus Material and Procedure

As online shopping is becoming more and more frequent among college students, we recruited 120 college students from a University in China as experimental subjects, and 113 subjects (53 males, *M*_*age*_ = 18.9, SD = 1.25) completed the study. The study used a 2 (product attribute: search attribute vs. experience attribute) × 2 (left-right position of graphic presentation: left image-right text vs. left text-right image) between-subjects design and participants were randomly assigned to one of the four scenarios.

The experimental stimuli include a mobile phone picture and text description. Specifically, the picture is selected from a real product picture of a shopping platform. The text descriptions were consistent with the pre-experiments, where it was verified that consumers could significantly distinguish between search and experience attributes. The left and right positions of the images and text were changed while the graphic and attributes remained consistent.

In order to control the influence of confounding factors on the study, the subjects were first asked about their knowledge of the phone, such as their knowledge of the camera function parameters and the screen parameters, to ensure that they could understand the information about the search attributes in the graphic. The participants were then shown a picture of the phone and a written description of the phone, and each group of participants was presented in turn with graphic information about the appearance and camera functions, and told to imagine that they were buying a new phone on a shopping platform. After reading the experimental material, participants reported their own product evaluation questions. Product evaluation involves two aspects: product appearance evaluation and product function evaluation measured by seven-point Likert scales involving interest, quality, trust, support, and usefulness (Peracchio and Tybout, 1996). Specifically, the evaluation of appearance includes 5 items: “I am interested in the appearance of this phone,” “I think the screen of this phone is comfortable to use,” “I favor the appearance design of this phone,” “I think the quality of this phone’s casing is good,” and “I trust the quality of this phone’s casing” (1 = strongly disagree, 7 = strongly agree). And the evaluation of function also includes 5 items: “I like the photo function of this phone,” “I think the picture quality of this phone is clear,” “I think the photo function of this phone is trustworthy,” “I think the photo function of this phone is valuable,” and “I believe I am satisfied with the photos taken by this phone” (1 = strongly disagree, 7 = strongly agree). Finally, subjects were also asked to report demographic variables such as gender and age.

#### Results

**FIGURE 2:**
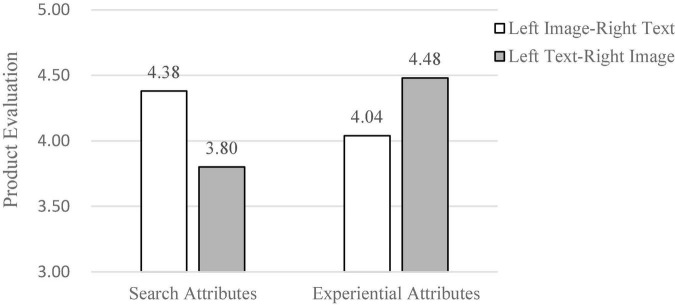
The ANOVA result (Study 1).

We conducted an ANOVA analysis using the graphical left and right position presentation and product attributes as independent variables and the product evaluation (α = 0.82) as dependent variable. The results showed that there was a significant interaction effect on product evaluation between product attributes and left-right position of the graphic [*F*(1, 109) = 7.06, *p* = 0.02] (as shown in Figure 2). Specifically, for the search attributes, compared to the presentation of the left text-right image (*M*_*left text–right image*_ = 3.80, SD = 1.19), the presentation of the left image−right text (*M*_*left image–right text*_ = 4.38, SD = 0.97) led to higher product valuation, *F*(1, 56) = 2.93, *p* = 0.04, *Cohen’s d* = 0.53.

Whereas when consumers were presented with product descriptions of experience attributes, compared to a left image-right text presentation (*M*_*left image–right text*_ = 4.04, SD = 0.77), a left text-right image presentation (*M*_*left text–right image*_ = 4.48, SD = 1.07) led consumers to higher ratings of product appearance. The effect was marginally significant [*F*(1, 53) = 2.15, *p* = 0.06, *Cohen’s d* = 0.47]. The results of the photographic function evaluations showed a similar pattern (*M*_*left image–right text*_ = 4.12, SD = 0.80; *M*_*left text–right image*_ = 4.50, SD = 0.91), *F*(1, 53) = 2.29, *p* = 0.06, *Cohen’s d* = 0.45. Gender and age had no significant effect on the dependent variable. These findings support our prediction that the interaction effect of product attributes and the left-right position of the graphic can significantly influence participants’ product evaluations. Thus, the results support H1 and H2.

#### Discussion

The results of Study 1 provide a preliminary test of H1 and H2, and are supported by the data. When presenting the search attributes of a product, the left image-right text presentation improved product ratings; when presenting the experience attributes of a product, the left text-right image presentation improved product ratings. However, Study 1 simply verified the occurrence of the phenomenon without exploring its essence, so we conducted Study 2 to examine the stability of the results and to verify the mediation mechanism behind the interaction between product attributes and left-right graphical presentation.

### Study 2

This study suggests that when product attributes and graphic positions are presented to match, consumers’ information processing fluency is enhanced, thus contributing to higher product evaluation. In order to test this hypothesis, we conducted Study 2. Additionally, to demonstrate the generalizability of our findings, we chose a gray sweater used for a class party as the material for our experiment, we chose a gray jumper for a class party as the experimental material, where the gray jumper is a unisex garment and gender differences can be excluded.

#### Pre-test

In order to ensure that the manipulation of the experimental materials in the formal study was feasible, we used the same procedures as the pre-study in Study 1. In this case, the text descriptions were chosen as stimulus materials for the sweatshirt fabric and the appearance, respectively. The text descriptions used in the pre-experiment and the main study are shown in Table 1.

The results of the pre-study showed that the 77 participants perceived product attributes differed significantly (41 males, *M*_*age*_ = 23.9, SD = 1.69), and independent samples *t*-tests showed that participants perceived significantly higher scores for the experience attribute of the sweatshirt fabric than for the search attribute (*M*_*search*_ = 2.24, SD = 1.36; *M*_*experience*_ = 4.23, SD = 0.93), *F*(1,75) = 4.20, *p* = 0.03, *Cohen’s d* = 1.71. And participants’ perceived overall effects also differed significantly between the search and experience attributes (*M*_*search*_ = 2.18, SD = 1.31; *M*_*experience*_ = 3.92, SD = 1.06), *F*(1, 75) = 4.50, *p* = 0.03, *Cohen’s d* = 1.46. Higher scores indicate that consumers perceive the product attribute to be on the experiential side, while lower scores indicate that it is on the search side. In summary, the experimental material manipulation of the gray sweatshirt was successful.

#### Stimulus Material and Procedure

This study used a 2 (product attribute: search attribute vs. experience attribute) × 2 (left-right position of graphic: left image-right text vs. left text-right image) between-subjects design. The study was conducted at a university in Guangzhou, China. 120 students were recruited, and 113 subjects eventually completed the study (56 males, *M*_*age*_ = 20.3, SD = 0.89). We first measured participants’ experience of buying clothes online, the factors they valued and their knowledge of clothing fabrics, with the aim of increasing their immersion when viewing product information, as well as ensuring that they understood the product attribute information in the illustrations.

At the outset subjects were told to participate in a simulated online purchase of class clothing, with images taken from practice in a real e-commerce situation, where the fabric of the sweatshirt was represented by a selection of three images showing details of the sweatshirt, and the overall effect was represented by a selection of images of a complete hooded sweatshirt being worn. Participants were then asked to complete the information processing fluency scale with four 7-points Likert items adapted from Graf et al. (2018): “I think the graphic presentation of information on this sweatshirt is clear and crisp,” “It takes a lot of effort to understand the information,” “It is easy to process the information,” and “It is easy to imagine the detailed design” (1 = not at all, 7 = very much) (Graf et al., 2018). The scales related to product evaluation were the same as in Study 1. Finally, participants filled in the relevant demographic variables.

#### Results

**FIGURE 3:**
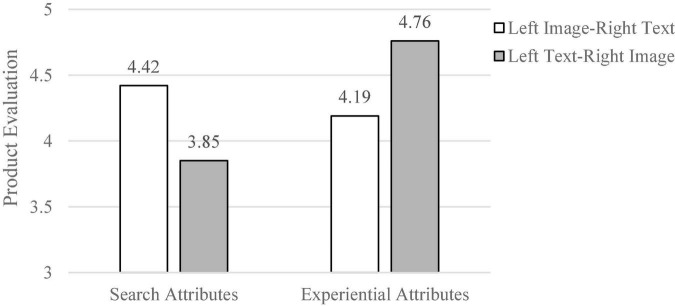
The ANOVA result (Study 2).

The analysis was carried out with graphic left-right position presentation and product attributes as independent variables and product detail evaluation (α = 0.79) and effect presentation evaluation (α = 0.75) as dependent variables. The results of the ANOVA analysis showed that the interaction terms presented in the left and right positions of the product attributes and graphics had a significant effect on product detail ratings [*F*(1, 109) = 6.01, *p* = 0.02] (as shown in Figure 3). Specifically, when consumers were presented with product descriptions of search attributes, the left image-right text presentation resulted in higher product ratings relative to the left text-right image presentation [*M*_*left image–right text*_ = 4.42, SD = 0.64, *M*_*left text–right image*_ = 3.85, SD = 0.69, *F*(1, 55) = 2.77, *p* = 0.04, *Cohen’s d* = 0.87]. When presented with the experience attributes, the left text-right image presentation order resulted in higher product evaluations relative to the left image-right text presentation order (*M*_*left image–right text*_ = 4.19, SD = 0.80, *M*_*left text–right image*_ = 4.76, *SD* = 0.87), *F*(1, 55) = 2.93, *p* = 0.04, *Cohen’s d* = 0.69. Gender and age had no significant effect on the dependent variable. In summary, both H1 and H2 are supported by the data.

In order to examine the mediating role of information processing fluency, we conducted a Bootstrap test based on the mediation analysis procedure proposed by Zhao et al. (2010), with a sample size of 5000, with reference to the moderated mediation analysis model (Model 8). The mean value of the interaction effect size was 0.2118 and the 95% confidence intervals were (0.0500, 0.5012), respectively, with the interval not containing 0, indicating the presence of a moderating mediating effect. Specifically, the mean value of the indirect effect size was −0.1107 when the product attribute was a search attribute, and the 95% confidence intervals for the Bootstrap test were (0.3207, −0.0033), respectively, with the intervals not containing 0, indicating a significant indirect effect. In other words, a mediating effect of information processing fluency exists when presenting search attributes. Comparatively, when presenting the experience attribute, the mean indirect effect size was 0.1012 and the 95% confidence intervals for the Bootstrap test were (0.0130, 0.2477), respectively, with the interval not containing 0, indicating a significant indirect effect. A mediating effect of information processing fluency exists when presenting experience attributes. The results of the effect presentation evaluation show a similar pattern. This suggests that both our H3a and H3b are supported by the data.

#### Discussion

The empirical results of Study 2 show that the findings of Study 1 still emerge when the product category is changed. The findings from Study 2 also provide insights into the mediating mechanisms by which online advertising influences product evaluation. When graphic information is presented in a way that matches consumers’ needs of psychological closure, it can increase consumer information processing fluency, which in turn influences consumers’ product evaluations. The positive effects of graphic location effects and product attributes on product evaluation (H1 and H2) and the mediating role of information processing fluency (H3) are demonstrated.

### Study 3

The purpose of Study 3 was to (i) test the robustness of the findings, and (ii) examine whether the findings could be applied to more generalized consumption scenarios: extending to another product category. Study 3 used a 2 (product attribute category: search attribute vs. experience attribute) × 2 (graphic-text position: left text-right image vs. left image-right text) between-subject design.

#### Stimulus Materials and Procedures

Referring to Jain and Posavac’s (2001) study, we chose mountain bikes as the experimental product. Specifically, we selected product images and attribute feature descriptions from a real shopping platform. The search attributes were mainly related to specific size, material, color and other characteristics, while the experience attributes emphasized the effectiveness of using the bicycle (as shown in Table 1). To avoid the influence of branding factors on the experimental effects, Study 3 used a virtual brand. As in previous studies, Study 3 also conducted a pre-test experiment on how the attribute categories were manipulated, and the results were shown to be successful.

The study subjects were undergraduate students from a university in Guangzhou, China, and the subjects were paid ¥20 after completing the experiment. The subjects were told to participate in a product testing activity for a brand. Upon arrival at the laboratory, subjects were randomly assigned to four experimental groups. After reading the experimental materials, subjects would complete product evaluations based on their favorites. Product evaluations were measured in the same way as in Study 1. Finally, subjects were also asked to provide information such as gender and age.

#### Results

**FIGURE 4:**
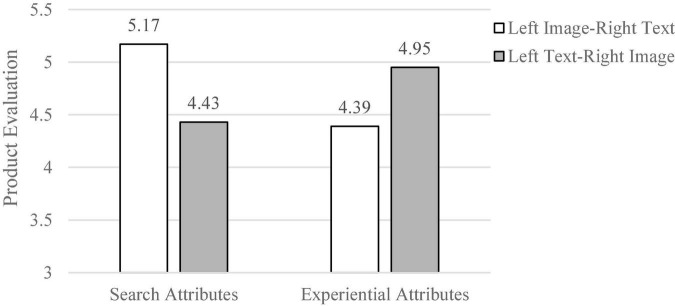
The ANOVA result (Study 3).

A total of 116 subjects were recruited for Study 3, 52 of whom were male, with a mean age of 20.4 years and a standard deviation of 0.98. Study 3 product evaluations (α = 0.89) were subjected to a two-factor (product attribute category × graphic location) ANOVA analysis. The results of the analysis revealed a high significant level of interaction between product attribute category and graphic location on product evaluation [*F*(1,112) = 12.31, *p* = 0.08] (as shown in Figure 4). Specifically, when presenting with search attributes, the left image-right text presentation stimulated higher product evaluations compared to the left text-right image presentation (*M_*left*_ _*image–right*_ _*text*_* = 5.17, SD = 0.94; *M_*left text*–right image_* = 4.43, SD = 1.03), *F*(1, 54) = 4.74, *p* = 0.03, *Cohen’s d* = 0.77; however, when presenting with experience attributes, the left text-right image presentation stimulated higher product evaluations compared to the left image-right text presentation (*M_*left*_ _*image–right*_ _*text*_* = 4.39, SD = 0.98; *M_*left text*–right image_* = 4.95, SD = 0.99), *F*(1, 58) = 4.70, *p* = 0.03, *Cohen’s d* = 0.58. In addition, age and gender did not have a significant effect on product evaluation. Taken together, the research hypotheses 1 and 2 were again supported by the data.

#### Discussion

In Study 3, we used a completely different product category from the previous study and again verified Hypotheses 1 and 2. This suggests that the findings of this paper are better robust and can be applied to a wider range of product categories.

## Discussion and Conclusion

Online shopping has become an indispensable way of consumption, and the major online shopping platforms have opened up a social e-commerce model in order to break through and grow the number of users, extending the target audience to townships and overseas. However, due to the lack of direct touch experience in online shopping, consumers need to rely on online visual information to make product evaluation and purchase judgments. Research on visual information in e-commerce has been extensively found in the consumer domain, such as examining the impact of image context (Yoo and Kim, 2014), image content (Aydınoğlu and Cian, 2014), and information presentation (Kim and Lennon, 2008) on consumers. However, there is a gap in the research on the impact of images and text together and the placement of both on consumers as the most basic form of information delivery. We wanted to understand which visual information presentation method was more appropriate for different product attributes. Therefore, this study investigates the possibility that different graphic placements have an impact on consumer evaluation.

Three studies were designed and conducted to investigate the effects of product information type (search attribute and experience attribute) and presentation order (left text-right image and left image-right text) on product evaluation. Specifically, Study 1 investigated the interactive effect of product information type and presentation order on product evaluation; Study 2 re-examined the effect of product information type and presentation order and tested the mediating effect of information processing fluency. To ensure the robustness of our findings, we conducted Study 3. We found that for the search attribute, the left text-right image presentation increased information processing fluency compared to the left text-right image presentation, which in turn improved consumers’ product evaluation; however, for the experience attribute, the left text-right image presentation increased information processing fluency compared to the left text-right image presentation, which in turn improved consumers’ product evaluation. In turn, it can improve consumers’ product evaluation.

Consumers unconsciously create a need for psychological closure when processing information, as evidenced by the desire to finish thinking about the information at the end of their browsing (Li et al., 2010). Due to writing and reading habits, it is common to think that things start on the left and end on the right. In this case, we believe that placing abstract information on the left and concrete information on the right can help people to collect their divergent thoughts and satisfy the need for cognitive closure. Such reading habits are compatible with consumers’ mental representations. When people are confronted with products that match their value preferences, they are more likely to stimulate their representations of habits and goals, and thus process product-related information fluently (Jin and Zhu, 2016). Based on the fact that processing fluency is a subjective feeling of ease or difficulty in evaluating objects when consumers process information, and that high fluency can trigger positive emotions in individuals when processing information and serve as an important cue for evaluating processing objects. It is reasonable to believe that processing fluency can be increased when the visual information presentation model meets the consumer’s need for cognitive closure, as it matches the consumer’s mental representation. The higher the processing fluency, the more positive the subsequent evaluation of the object being processed (Schwarz, 2004).

### Implications

This paper contributes to the previous research in two main ways. Firstly, most previous studies on product evaluation and purchase intention in online shopping contexts have focused on factors such as the order of presentation of different types of product images (Huang et al., 2016), image context (Yoo and Kim, 2014), online reviews (Kronrod and Danziger, 2013), and shop design, with little research on the synergistic effects of product information text and images. There is little research on the synergy of product information text and images. This paper looks at a more common phenomenon, combining different horizontal placement orders to examine the combined impact of images and text.

Secondly, this paper builds on previous literature examining product attributes by introducing a comparison between images and the two attributes text to analyze the relationship between the three from an abstract and concrete perspective. Previous research has found differences in the level of interpretation between pictures and text when they present the same content. Often the images displayed are so similar to the real product that people tend to analyze the product in the image with the perception of the real product, creating a closer psychological distance between the consumer and the product. Reading textual information, on the other hand, creates a sense of distance between people and the event (thing) being described (Coulmas, 2003). The further (closer) the psychological distance, the higher (lower) the level of interpretation and the more abstract (concrete) it is (Liberman and Trope, 1998). However, when images and text are presented with different content, the abstract-concrete relationship between the two changes and consumers judge the attributes of the displayed product based on the textual information. When presenting product information, online merchants can consider whether to choose left image-right text or left text-right image in relation to the product attributes. In future management practice, this can also be extended to a wider range of areas, such as product packaging, print advertising design and multimedia applications, where the position of images and text can be rationalized according to the information to be displayed.

In conclusion, our study is a useful addition to the theory of psychological closure, validating the phenomenon in a wider practical context. It also provides certain managerial insights. For example, placing content that allows for divergent thinking on the left and convergent thinking on the right, depending on the relationship between pictures and text, can help to improve people’s understanding of the information.

### Limitations

On the one hand, the present study, which is based on left-right position, needs to take into account the subjects’ responses to left-right position. For example, participants could be asked whether they are left- or right-handed, and this factor needs to be taken into account, as well as writing and reading habits. It is not yet known whether the same phenomenon will occur when people shop online in different reading cultures. On the other hand, there are two ways of processing textual and photographic information: scanning and photographic, which were not considered in the experimental design and need to be taken into account in future studies.

## Data Availability Statement

The original contributions presented in the study are included in the article/Supplementary Material, further inquiries can be directed to the corresponding author.

## Ethics Statement

The studies involving human participants were reviewed and approved by the School of Management, Jinan University, China. The patients/participants provided their written informed consent to participate in this study.

## Author Contributions

All authors listed have made a substantial, direct, and intellectual contribution to the work, and approved it for publication.

## Conflict of Interest

The authors declare that the research was conducted in the absence of any commercial or financial relationships that could be construed as a potential conflict of interest.

## Publisher’s Note

All claims expressed in this article are solely those of the authors and do not necessarily represent those of their affiliated organizations, or those of the publisher, the editors and the reviewers. Any product that may be evaluated in this article, or claim that may be made by its manufacturer, is not guaranteed or endorsed by the publisher.

## References

[B1] Avnet T. Higgins E. T. (2006). How regulatory fit affects value in consumer choices and opinions. *J. Mark. Res.* 43 1–10. 10.1509/jmkr.43.1.1 11670861

[B2] Aydınoğlu N. Z. Cian L. (2014). Show me the product, show me the model: effect of picture type on attitudes toward advertising. *J. Consum. Psychol.* 24 506–519. 10.1016/j.j.2014.04.002

[B3] Beike D. Wirth-Beaumont E. (2005). Psychological closure as a memory phenomenon. *Memory* 13 574–593. 10.1080/09658210444000241 16076673

[B4] Beike D. R. Adams L. P. Wirth-Beaumont E. T. (2007). Incomplete inhibition of emotion in specific autobiographical memories. *Memory* 15 375–389. 10.1080/09658210701276850 17469018

[B5] Blankson C. Kalafatis S. P. (1999). Issues and challenges in the positioning of service brands: a review. *J. Prod. Brand Manage*. 8 106–118. 10.1108/10610429910266968

[B6] Chae B. Hoegg J. (2013). The future looks “right”: effects of the horizontal location of advertising images on product attitude. *J. Consum. Res.* 40 223–238. 10.1086/669476

[B7] Chatterjee A. (2001). Language and space: some interactions. *Trends Cogn. Sci.* 5 55–61. 10.1016/S1364-6613(00)01598-9 11166635

[B8] Chatterjee A. Southwood M. H. Basilico D. (1999). Verbs, events and spatial representations. *Neuropsychologia* 37 395–402. 10.1016/S0028-3932(98)00108-0 10215086

[B9] Christman S. Pinger K. (1997). Lateral biases in aesthetic preferences: pictorial dimensions and neural mechanisms. *Laterality* 2 155–175. 10.1080/713754266 15513061

[B10] Coulmas F. (2003). *Writing Systems: An Introduction to their Linguistic Analysis.* Cambridge: Cambridge University Press.

[B11] Dechesne M. Kruglanski A. W. (2004). “Terror’s epistemic consequences: existential threat and the quest for certainty and closure,” in *Handbook of Experimental Existential Psychology*, eds Greenberg J. Koole S. L. Pyszczynsk T. (New York, NY: Guilford Press).

[B12] Finke R. A. (1995). “Creative insight and preinventive forms,” in *The Nature of Insight*, eds Sternberg R. J. Davidson J. E. (Cambridge, MA: The MIT Press), 255–280.

[B13] Forceville C. (2002). The identification of target and source in pictorial metaphors. *J. Pragmat.* 34 1–14. 10.1016/S0378-2166(01)00007-8

[B14] Ford G. T. Smith D. B. Swasy J. L. (1990). Consumer skepticism of advertising claims: testing hypotheses from economics of information. *J. Consum. Res.* 16 433–441. 10.1086/209228

[B15] Graf L. K. Mayer S. Landwehr J. R. (2018). Measuring processing fluency: one versus five items. *J. Consum. Psychol.* 28 393–411. 10.1002/jcpy.1021

[B16] Hirschman E. C. (1986). Humanistic inquiry in marketing research: philosophy, method, and criteria. *J. Mark. Res.* 23 237–249.

[B17] Hoegg J. Alba J. W. Dahl D. W. (2010). The good, the bad, and the ugly: influence of aesthetics on product feature judgments. *J. Consum. Psychol.* 20 419–430. 10.1016/j.jcps.2010.07.002

[B18] Houston M. J. Childers T. L. Heckler S. E. (1987). Picture-word consistency and the elaborative processing of advertisements. *J. Mark. Res.* 24 359–369. 10.1177/002224378702400403

[B19] Huang J. Guo Y. Xiong X. Wang Y. (2016). Research on the Influence of Online Picture Presentation Order on Consumers’ Purchase Intention —— from the perspective of information processing mode. *J. Mark. Sci.* 12 51–69.

[B20] Huang P. Lurie N. H. Mitra S. (2009). Searching for experience on the web: an empirical examination of consumer behavior for search and experience goods. *J. Mark.* 73 55–69. 10.1509/jmkg.73.2.55 11670861

[B21] Huang J. Zou Y. P. Liu H. L. Wang J. T. (2017). Is “Dynamic” Better Than “Static”? The Effect of Product Presentation on Consumers’ Evaluation—The Mediation Effect of Cognitive Processing. *Chin. J. Manag.* 14, 742–750.

[B22] Jain S. P. Posavac S. S. (2001). Prepurchase attribute verifiability, source credibility, and persuasion. *J. Consum. Psychol.* 11 169–180. 10.1207/S15327663JCP1103_03 26627889

[B23] Jin F. Zhu H. (2016). Consumers’ sense of power and impulse buying. *Acta Psychol. Sin.* 48 880–890. 10.3724/SP.J.1041.2016.00880

[B24] Kim J. (2017). The influence of graphical versus numerical information representation modes on the compromise effect. *Mark. Lett.* 28 397–409.

[B25] Kim M. Lennon S. (2008). The effects of visual and verbal information on attitudes and purchase intentions in internet shopping. *Psychol. Mark.* 25 146–178. 10.1002/mar.20204

[B26] Kivetz R. Urminsky O. Zheng Y. (2006). The goal-gradient hypothesis resurrected: purchase acceleration, illusionary goal progress, and customer retention. *J. Mark. Res.* 43 39–58. 10.1509/jmkr.43.1.39 11670861

[B27] Kronrod A. Danziger S. (2013). Wii will rock you!” The use and effect of figurative language in consumer reviews of hedonic and utilitarian consumption. *J. Consum. Res.* 40 726–739. 10.1086/671998

[B28] Lancaster F. W. (1971). The cost-effectiveness analysis of information retrieval and dissemination systems. *J. Am. Soc. Inf. Sci.* 22 12–27. 10.1002/asi.4630220104

[B29] Lee A. Y. Labroo A. A. (2004). The effect of conceptual and perceptual fluency on brand evaluation. *J. Mark. Res.* 41 151–165. 10.1509/jmkr.41.2.151.28665 11670861

[B30] Li X. Wei L. Soman D. (2010). Sealing the emotions genie: the effects of physical enclosure on psychological closure. *Psychol. Sci.* 21 1047–1050. 10.1177/0956797610376653 20622143

[B31] Liberman N. Trope Y. (1998). The role of feasibility and desirability considerations in near and distant future decisions: a test of temporal construal theory. *J. Pers. Soc. Psychol.* 75 5–18. 10.1037/0022-3514.75.1.5

[B32] Liu L. Yu C. Zhao P. (2018). The influence of graphic information on consumers’ interactive behavior and brand relationship. *Mark. Sci.* 31 90–100. 10.3969/j.issn.1672-0334.2018.01.007

[B33] Mitchell A. A. Olson J. C. (1981). Are product attribute beliefs the only mediator of advertising effects on brand attitude? *J. Mark. Res.* 18 318–332. 10.1177/002224378101800306

[B34] Mitchell V. W. Boustani P. (1994). A preliminary investigation into pre-and post-purchase risk perception and reduction. *Eur. J. Mark.* 28 56–71. 10.1108/03090569410049181

[B35] Mokhlis S. Yaakop A. Y. (2012). Consumer choice criteria in mobile phone selection: an investigation of Malaysian University students. *Int. Rev. Soc. Sci. Hum.* 2 203–212.

[B36] Nelson P. (1970). Information and consumer behavior. *J. Polit. Econ.* 78 311–329. 10.1086/259630

[B37] Noseworthy T. J. Cotte J. Lee S. H. (2011). The effects of ad context and gender on the identification of visually incongruent products. *J. Consum. Res.* 38 358–375. 10.1086/658472

[B38] Nunes J. C. Dreze X. (2006). The endowed progress effect: how artificial advancement increases effort. *J. Consum. Res.* 32 504–512. 10.1086/500480

[B39] Orús C. Gurrea R. Flavián C. (2017). Facilitating imaginations through online product presentation videos: effects on imagery fluency, product attitude and purchase intention. *Electron. Commer. Res.* 17 661–700. 10.1007/s10660-016-9250-7

[B40] Ouellet M. Santiago J. Funes M. J. Lupiánez J. (2010). Thinking about the future moves attention to the right. *J. Exp. Psychol. Hum. Percept. Perform.* 36 17–24. 10.1037/a0017176 20121292

[B41] Peracchio L. A. Tybout A. M. (1996). The moderating role of prior knowledge in schema-based product evaluation. *J. Consum. Res.* 23 177–192. 10.1086/209475

[B42] Peschel A. O. Orquin J. L. (2013). A review of the findings and theories on surface size effects on visual attention. *Front. Psychol.* 4:902. 10.3389/fpsyg.2013.00902 24367343 PMC3856423

[B43] Pieters R. Wedel M. (2004). Attention capture and transfer in advertising: brand, pictorial, and text-size effects. *J. Mark.* 68 36–50. 10.1509/jmkg.68.2.36.27794 11670861

[B44] Pieters R. Wedel M. Zhang J. (2007). Optimal feature advertising design under competitive clutter. *Manage. Sci.* 53 1815–1828. 10.1287/mnsc.1070.0732 19642375

[B45] Romero M. Biswas D. (2016). Healthy-left, unhealthy-right: Can displaying healthy items to the left (versus right) of unhealthy items nudge healthier choices? *J. Consum. Res.* 43 103–112. 10.1093/jcr/ucw008

[B46] Santiago J. Lupáñez J. Pérez E. Funes M. J. (2007). Time (also) flies from left to right. *Psychon. Bull. Rev.* 14 512–516. 10.3758/BF03194099 17874598

[B47] Schwarz N. (2004). Metacognitive experiences in consumer judgment and decision making. *J. Consum. Psychol.* 14 332–348. 10.1207/s15327663jcp1404_2

[B48] Sevilla J. Kahn B. E. (2014). The completeness heuristic: product shape completeness influences size perceptions, preference, and consumption. *J. Mark. Res.* 51 57–68. 10.1509/jmr.12.0153 11670861

[B49] Shepard R. N. (1967). Recognition memory for words, sentences, and pictures. *J. Verbal Learn. Verbal Behav.* 6 156–163. 10.1016/S0022-5371(67)80067-7

[B50] Spalek T. M. Hammad S. (2005). The left-to-right bias in inhibition of return is due to the direction of reading. *Psychol. Sci.* 16 15–18. 10.1111/j.0956-7976.2005.00774.x 15660846

[B51] Srinivasan S. S. Till B. D. (2002). Evaluation of search, experience and credence attributes: role of brand name and product trial. *J. Prod. Brand Manage.* 11 417–431. 10.1108/10610420210451616

[B52] Vallesi A. Binns M. A. Shallice T. (2008). An effect of spatial–temporal association of response codes: understanding the cognitive representations of time. *Cognition* 107 501–527. 10.1016/j.cognition.2007.10.011 18076872

[B53] Voss K. E. Spangenberg E. R. Grohmann B. (2003). Measuring the hedonic and utilitarian dimensions of consumer attitude. *J. Mark. Res.* 40 310–320. 10.1509/jmkr.40.3.310.19238 11670861

[B54] Ward T. B. (1995). “What’s old about new ideas,” in *The Creative Cognition Approach*, eds Smith S. M. Ward T. B. Finke R. A. (Cambridge, MA: MIT Press), 157–178. 10.1177/0894318416630096

[B55] Winkielman P. Cacioppo J. T. (2001). Mind at ease puts a smile on the face: psychophysiological evidence that processing facilitation elicits positive affect. *J. Pers. Soc. Psychol.* 81 989–1000. 10.1037/0022-3514.81.6.989 11761320

[B56] Wright A. A. Lynch J. G. Jr. (1995). Communication effects of advertising versus direct experience when both search and experience attributes are present. *J. Consum. Res.* 21 708–718. 10.1086/209429

[B57] Wyer R. S. Jr. Adaval R. (2009). “Social psychology and consumer psychology: an unexplored interface,” in *Social Psychology of Consumer Behavior*, ed. Wänke M. (New York, NY: Psychology Press), 19–61.

[B58] Xia H. Pan X. Zhou Y. Zhang Z. J. (2020). Creating the best first impression: designing online product photos to increase sales. *Decis. Support Syst.* 131:113235. 10.1016/j.dss.2019.113235

[B59] Yoo J. Kim M. (2014). The effects of online product presentation on consumer responses: a mental imagery perspective. *J. Bus. Res.* 67 2464–2472. 10.1016/j.jbusres.2014.03.006

[B60] Zhao X. Lynch Jr J. G. Chen Q. (2010). Reconsidering Baron and Kenny: Myths and truths about mediation analysis. *J. Consum. Res*. 37, 197–206.

